# The Battle of Jutland Film

**Published:** 1921-09-17

**Authors:** H. Rowson

**Affiliations:** 76-78 Wardour Street, W. 1. Managing Director, Ideal Films, Ltd.


*To the Editor of* The Hospital.

### The Battle of Jutland Film.

*To the Editor of* The Hospital.

Sir,—A number of cinema proprietors, who are about to show above picture, are desirous of engaging the esrvices of Jutland survivors who are willing to speak to the film when it is being exhibited.

As proprietors of the picture in question, we are prepared to make a register of all such men, if they will be good enough to send us their names and addresses as well as their rank and the name of their ship.

We will then put them in touch, in each case, with the nearest cinemas showing the picture.

As this is the only means we have of reaching the survivors of the battle, we shall esteem it a great favour if you would be so good as to grant us the courtesy of your columns. We beg to remain, your faithfully,

H. Rowson,
Managing Director,
Ideal Films, Ltd.,

76-78 Wardour Street, W. 1.
September 12, 1921.

